# The value and impact of health technology assessment: discussions and recommendations from the 2023 Health Technology Assessment International Global Policy Forum

**DOI:** 10.1017/S0266462323002763

**Published:** 2023-12-22

**Authors:** Rebecca Trowman, Antonio Migliore, Daniel A. Ollendorf

**Affiliations:** 1 Health Technology Assessment (HTAi), Perth, WA, Australia; 2 Health Technology Assessment International (HTAi), Edmonton, AB, Canada; 3 Tufts Medical Center, Center for the Evaluation of Value and Risk in Health, Boston, MA, USA

**Keywords:** technology assessment, biomedical, decision making, health care quality, access, and evaluation, quality assurance, health care, program evaluation

## Abstract

Health technology assessment (HTA) programs inform decision making about the value and reimbursement of new and existing health technologies; however, they are under increasing pressure to demonstrate that they are a cost-effective use of finite healthcare resources themselves. The 2023 HTAi Global Policy Forum (GPF) discussed the value and impact of HTA, including how it is assessed and communicated, and how it could be enhanced in the future. This article summarizes the discussions held at the 2023 HTAi GPF, where the challenges and opportunities related to the value and impact of HTA were debated. Core themes and recommendations identified that defining the purpose of value and impact assessment is an essential first step prior to undertaking it, and that it can be done through the use and expansion of existing tools. Further work around aligning HTA programs with underlying societal values is needed to ensure the long-term value and impact of HTA. HTA could also have a role in assessing the efficiency of the wider health system by applying HTA methods or concepts to broader budgetary allocations and organizational aspects of health care. Stakeholders (particularly patients, industry, and clinicians but also payers, wider society, and the media) should ideally be actively engaged when undertaking the value and impact assessment of HTA. More concerted efforts in communicating the role and remit of HTA bodies would also help stakeholders to better understand the value and impact of HTA, which in turn could improve the implementation of HTA recommendations and application to future actions in the lifecycle of technologies.

## Introduction

Health technology assessment (HTA) programs often inform decision making about the value and reimbursement of new and existing tests or treatments; however, they are under increasing pressure to demonstrate that they are a cost-effective use of finite resources themselves. In other words, demonstrated gains in population health should be optimized given the budget available for HTA (1). Evidence of the value and impact of HTA programs is of strategic importance to increase legitimacy within healthcare systems and potentially defend against funding cuts or challenges such as in times of political change where the support for HTA may be questioned. In the current climate, HTA is potentially at risk of being perceived as an unnecessary barrier or hurdle to access for innovative treatments (2). Given the increasing pace of innovation (e.g., cell and gene therapies) and the continued efforts by regulatory authorities to accelerate drug and device approvals, there can be a perceived need to make adoption and reimbursement decisions more quickly; meaning that the future of HTA may be under threat without appropriate action.

The topic under discussion therefore relates to two distinct, but inter-related dimensions: value and impact. The 2023 HTAi Global Policy Forum (GPF) background paper (3) sets out the following definitions for the purposes of the GPF discussion:

- *Value*: The perceived worth or benefit of HTA, which may vary according to stakeholder type, local setting, and other factors.
- *Impact:* Qualitative and/or quantitative assessment or review of the effects of HTA, which may vary by perspective, setting, and other factors, and which may include *valuation* exercises. Impacts can be considered as direct, indirect, and/or intangible.

**Figure 1.**
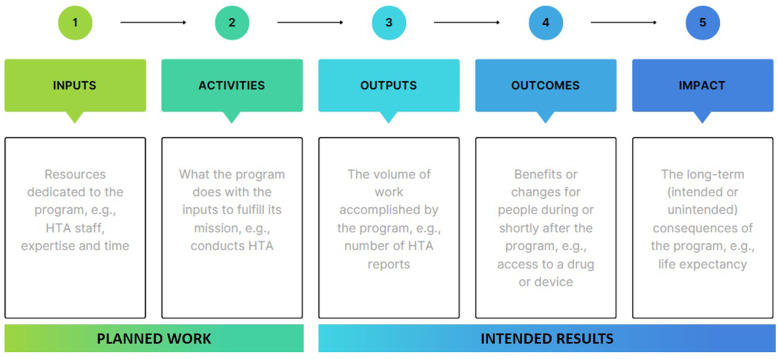
Program logic model of health technology assessment (HTA). Adapted from Harris et al. (4).

To help visualize the above definitions, a conceptual framework that represents the process of HTA and highlights how the terms were defined in the discussions is presented in Figure 1. This conceptual framework is based on a “logic model”; a graphic which represents the theory of how an intervention produces its output, outcomes, and impacts based on inputs and related activities. The value of each of these resulting elements, including impact, can be defined and measured quantitatively or qualitatively.

As noted in the definition of the discipline, HTA is a process that can include activities such as clinical and economic assessment and ethical, social, and organizational assessments. Its purpose is to “inform decision-making to promote an equitable, efficient and high-quality health system” (5).

Many countries today use HTA as a policy tool to help health systems determine the best use of finite health resources for investments in new technologies, and HTA recommendations can be mandated within health systems and used to facilitate pricing and reimbursement. There are jurisdictions, however, where HTA recommendations are not mandated and so HTA bodies instead aim to prospectively shape health care (e.g., in the US, Canada, and countries in Latin America); further, there are pluralistic health systems with multiple payers or sectors where a single HTA and accompanying recommendation may not meet the needs of all decision makers (6).

Considering the remit of a given HTA body, and the health care budgets employed within a health system are essential elements for determining the impact that HTA may have in specific settings. The ultimate value of HTA in a health system therefore may depend, in part, on its contribution to improved health status, reduced inequities, and increased efficiencies as well as contributions to a sustainable health system (7). One of the most important factors influencing the impact of HTA reports is arguably the directness of the relationship between a HTA body, policy and decision makers (8). Other factors influencing the impact that HTA recommendations may have include the level of available evidence (that can inform the HTA recommendations), resourcing and staffing constraints, the willingness and ability of stakeholders to engage in HTA processes, communication and uptake of HTA reports and recommendations (9). The ability of the HTA process to “keep pace” with medical innovations is also noted as important to both maintain the impact of HTA and its perceived value to many stakeholder groups.

Empirical evidence of the impact of HTA on either health outcomes or spending is relatively scarce. Much of the existing literature has tended to focus on the outputs of HTA and the uptake of its recommendations by decision makers. Some commonly used quantitative metrics, frameworks, and other qualitative and more conceptual metrics of the impact of HTA and the subsequent value from this are described in the GPF background paper (3), including a review of current activities undertaken by multiple HTA bodies (see background paper). Other impacts of HTA that are more nuanced and not as easily quantified include increasing transparency, building stakeholder dialogue and trust, fostering innovation and mindset shifts (i.e., making decisions based on evidence instead of other factors such as favoritism or activism).

There are of course challenges in assessing the value and impact of HTA; it is both time consuming and resource intensive. In 2020, the International Network of Agencies for Health Technology Assessment (INAHTA) conducted a two-part study that first aimed to determine what impact assessment activities are currently being undertaken by its members (10), and secondly identified the factors that enable or inhibit impact assessment (11). A lack of qualified staff, standardized tools or methods, financial or organizational resources, staff motivation (e.g., wanting to, or the feeling of having to, move onto the next assessment, rather than review the impacts of existing HTA reports) and suboptimal integration of the results of the impact assessment into everyday activities were cited as major barriers. The main aim of the 2023 HTAi GPF was to discuss the challenges, opportunities, and potential next steps pertaining to the assessment of the value and impact of HTA. This included considering why and how the value and impact should be assessed and also how it can be communicated, and how the value and impact of HTA can be enhanced moving forward.

## GPF meeting structure

Over 26–28 March 2023, eighty-four representatives from not-for-profit organizations (public HTA bodies, payers, and health systems) and for-profit organizations (pharmaceutical, biotech, and device companies), patient representatives, invited speakers, and HTAi leadership met in The Hague, The Netherlands to discuss these issues. The meeting was conducted under the Chatham House Rule (12), whereby participants are free to share information obtained at the meeting, but they may not reveal the identity or affiliation of the person providing the information. This article presents the authors’ view on the 2023 GPF and is not a consensus or official statement from individuals who attended the meeting or their organizations.

The GPF began with a keynote presentation that focused on a separate but related concept, “social pharmaceutical innovation” (13), with reference to the need for multi-stakeholder collaboration to ensure meaningful output – a relevant theme for HTA. This was followed by a debate on the value of HTA, focusing on whether the current methods are “fit-for-purpose.” Case studies and a panel session representing HTA bodies, regulators, patients, citizens, industry, and clinician perspectives were also presented to stimulate further debate on the key issues. The GPF members were then divided into smaller breakout groups to discuss the challenges, barriers, opportunities, and next steps related to six key themes, as described in Table 1.

**Table 1.** Key themes for the breakout groups session

1. What are the most important metrics of value and impact assessment? 2. How can stakeholders get engaged in assessing HTA value and impact as well as contributing to it? 3. What are the risks to HTA from incomplete, poorly communicated, or otherwise suboptimal evidence on value and impact? 4. How “valuable” is value and impact assessment? 5. How should the value and impact of HTA be communicated? 6. How can the value and impact of HTA be enhanced in the future?

## Meeting proceedings

The discussions from the 2023 GPF were wide-ranging, reflecting the broad nature of the topic selected. However, some repeated key themes emerged, which are summarized below.

### Defining the value and impact of HTA

The value and impact of HTA can be defined in a multitude of ways and quantification is challenging. Determining a useful counterfactual (i.e., what would happen in the same health system without HTA) is problematic due to the many contemporaneous changes that may have occurred in the health system after HTA’s inception and the fact that technologies may be implemented irrespective of the work of a HTA body (14). It was noted, however, that retrospective reviews of the anticipated health outcomes (as estimated through the HTA process) compared with the actual health outcomes could be considered as a possible proxy for a counterfactual, particularly in areas where longer-term data may now be available.

Furthermore, value and impact assessment can have many uses, from informing internal process and methods improvement to reporting toward continued funding or submitting business cases for additional funding (15). Therefore, what is measured, when, with what methods, and by whom will all be influenced by the purpose and audience for such assessments. The perspective that the value is assessed from is also an important *a priori* consideration. For example, a patient may place greater value on rapid access to innovative treatments without fully demonstrated clinical benefit, whereas a government, payer or even society as a whole, may place greater value on access to technologies that will maximize population health more broadly, with some patients potentially disadvantaged as a result (although it is acknowledged that this is a nuanced concept and there may be government and societal preferences toward technologies that may benefit disadvantaged groups). There are also examples of industry members who have developed and published statements supporting the value of HTA, an example of which was presented in the panel session (16).

The value and impact of HTA is also closely linked to the remit of the HTA body within its healthcare system. Clearly defining and understanding the remit that a HTA body has must occur before the value of the HTA activities can be determined (e.g., considering whether a HTA body must provide reimbursement advice on all new technologies within a specific timeframe from regulatory approval). The remit will also likely have a direct influence on how recommendations and reports from a HTA body are used and implemented (e.g., in systems where HTA recommendations are mandated) and this will have a substantial effect on the downstream impact and subsequent value of the HTA. It is possible that the greatest impact of HTA bodies comes when the HTA body is well integrated into the health system, so that the HTA recommendations are more likely to be effectively disseminated and implemented. Where this integration is less effective, stakeholder management with relevant stakeholders such as the public, providers, payers, and others, is often a cornerstone of the HTA process to facilitate adoption and implementation of final HTA recommendations (17).

### Measuring the value and impact of HTA

As noted, the value that an individual places on a particular outcome or impact may differ according to personal beliefs, cultures, and a range of possible other factors such as the overall health and wealth of a country. Therefore, value and impact assessment need to be relevant to the local context, disease or condition, and the perspective of the stakeholder(s) should be considered within the assessment.

Despite this, there are some general considerations pertaining to value and impact assessment. Firstly, the reason for conducting the assessment should be clear; for example, determining accountability of public funds and/or undertaking learning and process improvements. Secondly, tools such as the INAHTA impact framework (18;19) were noted as particularly useful. This framework has been adapted and utilized by a number of HTA bodies already. Such a framework could therefore serve as a starting point and could be expanded to include consideration of certain interdependencies such as interaction with regulatory authorities and other health sector stakeholders and decision makers (where relevant). Additionally, system level metrics and a checklist could be added to help apply the framework across multiple jurisdictions. Such developments could enable the INAHTA framework to be applied at a local (HTA body) context (as is the case now) and then used more widely to share learnings across jurisdictions.

GPF members further suggested that value and impact assessments should be done regularly to track progress, as part of a continuous learning cycle. However, it was noted that there should be flexibility in the timing of the assessments and that conducting assessments in a short-time frame would not allow for alterations and subsequent changes to be made and impacts felt. GPF members felt that 5-yearly increments would allow sufficient time to implement recommendations and process changes.

Finally, if HTA recommendations are not implemented, then it stands to reason that the full potential impact and subsequent value of the HTA recommendations on the health system will not be realized. This is not a new challenge as noted by Henshall et al. in 2002 (20); it has been observed that this aspect is one of the weakest elements of the HTA process. There needs to be greater effort by both the HTA bodies but also and other health system stakeholders such as policy and decision makers to carefully consider the feasibility of implementing the recommendations at a system level (with notable examples of how this is done by ZIN and Health Technology Wales discussed at the GPF and outlined on their respective web sites). These considerations should explore whether there are any supportive recommendations or actions that could facilitate implementation of HTA recommendations through closer consultation with key stakeholders (such as patients, clinicians – including medical societies who often develop clinical guidelines, payers, and health system managers) to increase the likelihood of successful implementation. Potential barriers to implementing HTA recommendations should be highlighted and explored. An example of such a challenge includes enforcing eligibility criteria for certain technologies that are not realistic for patients to achieve (e.g., patients who manage a condition primarily at home, but need a certain number of emergency department consultations to be considered eligible for a drug). Where technologies require system reorganization or behavioral changes that have not been captured within a clinical trial setting, then implementation may also need to be considered within the evaluation itself (21). Careful consideration of the HTA recommendations and whether they could unintentionally lead to health inequities (e.g., a technology being recommended after testing which may incur out of pocket costs for patients, or treatments being made available at centers not easily accessible for patients based remotely) is critical. Steps to ensure equitable access to all technologies (e.g., including patient accessibility as a domain of HTA particularly when considering technology implementation) are important for all HTA bodies and must be closely monitored and reviewed. Closer attention to the uptake of HTA recommendations with a qualitative review of any barriers or challenges to their uptake (as routinely conducted by Health Technology Wales) could also facilitate this process.

### The role of HTA in the wider health system

One of the key recurring points of discussion throughout the 2023 GPF concerned the current role of HTA in many countries, particularly in higher-income countries where HTA is increasingly a mechanism to aid reimbursement decisions for (typically new) drugs and devices. However, drugs and devices only account for 15–20 percent of a typical health budget. While some HTA bodies have a broader remit and look at procedures, vaccines, and other public health interventions, HTA concepts are not consistently applied at the broader health system level. GPF members argued that the value and impact of HTA could be greater and more widely recognized if the remit of HTA bodies, or at least the application of HTA principles, was extended to include broader organizational and budget allocation decisions. Examples were provided of countries with waitlists to see primary care physicians and other specialists that are considered unacceptable. Shortages of staff and beds, as well as limited resources for palliative care were also provided as examples that have not been subject to evidence-based decision making or principled allocation of resources. Without such an expansion of the HTA lens, there are likely to be broader funding issues that may prove catastrophic; for example, an increasing number of people dying on waiting lists each year. Expanding the remit of HTA to the broader heath system to include all aspects of healthcare provision and decision making may be warranted. HTA bodies may be able to see where more resources are needed to allow for the adoption of new technologies, however, this would require additional resources for HTA bodies to undertake and could be subject to factors outside of the scope of the health system.

### The role of societal values

One element that is critical in determining the broader value of HTA is considering the values and preferences of the society or the taxpayer – meaning patients, caregivers, citizens, and companies – it represents. Where HTA recommendations align with the values held by a society, then the ultimate value is likely to be higher with a probable greater uptake and implementation in the recommendations as a result. However, establishing societal preferences is not a widespread or straightforward activity (22), with many methodological challenges. Further exploration into democratic, deliberative decision making and methods to elicit societal values is needed to ensure that the values of the people that HTA is being conducted on behalf of are represented. Aligning assessment and appraisal of technologies to societal values, for example, prioritizing severity or reducing health inequities will require trade-offs and preferences to be elicited and how these factors are considered within a HTA should be reflective of these values and objectives and conducted transparently. Without this, HTA recommendations may be ignored, and people may ultimately lose trust in HTA decisions and bodies.

As previously mentioned, an additional element of elevating the value and impact of HTA may be to minimize health inequalities or, at the very least, not unintentionally exacerbated through HTA recommendations. More research is needed to determine the societal preferences around health inequities; that is, do different societies have a preference for minimizing gaps in health inequalities, or a preference to improve health outcomes universally, even if this extends gaps in health outcomes and how this trade off can be addressed (23). A white paper from the Institute for Clinical and Economic Review (ICER) on HTA methods and processes that may advance health equity was referenced during the GPF discussions (24). There was broad agreement that this is an area where further empirical research is needed, and this is one aspect where the impact of HTA could be increased.

### Engaging stakeholders

As with the HTA process itself, participation of stakeholders from the beginning of the assessment of the value and impact of HTA is important; however, identification of who should be involved is needed, and this will be context and assessment specific (25). Considering the perspectives of the “7Ps” (patients, providers, payers, purchasers, product makers, policy makers, and principal investigators) (26), plus the public and caregivers, is considered an appropriate base from which to begin, with associations representing these stakeholder groups as relevant. Additional “nontraditional” stakeholder groups such as judicial/legal systems and the media could also help increase the reach and therefore potential impact of HTA. Each stakeholder group will need different levels and methods of engagement, and those that are typically most engaged are the patients, providers (clinicians), and product makers (industry). Given the variety of stakeholder groups, a range of formats and messages should be developed and tailored to the interests, needs, and understanding of each audience. More proactive, rather than reactive, messaging about the value of HTA is also needed, with one key (sometimes overlooked) stakeholder being the media. Greater use of novel digital and online tools for communicating and engaging stakeholder groups (such as podcasts and social media) (27;28),may facilitate stakeholder engagement with the assessment of the impact of HTA.

### Resourcing value and impact assessment

It was acknowledged that value and impact assessment can be resource intensive and that resource constraints are only increasing with the number of emerging technologies and new requirements on HTA bodies (such as the EU HTA Regulation). Ensuring that HTA bodies have sufficient time, resources and expertise for conducting internal impact assessment activities is important as most HTA bodies are conducting their own assessment activities. This may, however, bias the results of the assessment (particularly if the results are tied to re-financing and future budgets) and may divert resources from core business. External assessments of value and impact assessment; undertaken by independent, neutral, and unbiased parties (e.g., national audit offices) could be an alternative approach, funded outside of the HTA body. The concept of sharing learnings in a resource-constrained environment could be expanded to include HTA methods and process training courses such as undertaken by organizations like the European Patients’ Academy on Therapeutic Innovation (EUPATI) (29). However, this would require the evaluation of such materials first to ensure that they also add value and have a positive impact on the HTA community.

### Communicating the value and impact of HTA

One area that was reported as challenging by nearly all GPF members was the communication of the value and impact of HTA. The purpose, methods, and processes of HTA can be perceived as overly complex, with often technical and opaque language used to describe it (30). HTA has essentially been created as a social construct to handle a basic ethical dilemma of making difficult decisions regarding the allocation of limited resources (31). This means that “negative” recommendations are inevitable, and this needs to be better explained to patients, the public, and others. Better communication of the work and outputs of HTA bodies is needed and the value proposition of HTA should be clearly articulated, including the notion that HTA is more than cost-effectiveness analysis and that different HTA bodies may look at the same evidence base and come to different conclusions. Describing the role of HTA in a post-pandemic setting is especially important as HTA was circumvented in some countries during the pandemic and may now be seen by some as an unnecessary hurdle. The role that HTA can and should play where regulatory decisions are made on scant evidence should be better explained.

Resourcing better communication is a challenge for many HTA bodies who are currently experiencing acute staff and skills shortages. Despite this, clearer communication is something that the HTA community can strive for. Use of unhelpful language, such as “rationing” and “cost-containment,” should be avoided and replaced with sensitive and respectful communication strategies. Further, there may be a role for organizations such as HTAi to assist in developing some core messages (e.g., around the value proposition of HTA). The risks of not adequately communicating the value and impact of a HTA body to all stakeholders may include legal challenges; problems in obtaining and/or sustaining funding; and loss of trust and reduced implementation of HTA recommendations. However, if the value and impact of HTA is communicated effectively, this could build trust, establish legitimacy, and ultimately increase the uptake and potential impact of HTA recommendations.

## Limitations

This article represents a summary of discussions held at the 2023 HTAi GPF. While this forum represents a broad range of views and perspectives, the membership of the GPF includes perspectives are from countries with established, mature HTA systems. While informants from beyond the GPF membership were approached for input to the background paper prior to the meeting, this is a limitation of the discussion summary as these views were not directly present at the GPF discussions. In low- and middle-income countries (LMICs), the value and impact of HTA may be greatest through increased transparency and enhanced legitimacy so that decisions around inclusion of a technology in an overall benefits package are not made at random or to favor those in power.

Further, the GPF primarily comprises HTA body representatives and life science industry organizations. Patient representatives are specifically consulted during the development of the background paper and are invited to the meeting to present and participate; however, some key stakeholder groups (such as clinicians, payers, and policy makers) are less well represented in the discussion.

## Recommendations and next steps

GPF participants were asked to identify what they considered to be the priority recommendations for action arising from the meeting. From this exercise, two recommendations were highlighted:

1. Development of a “HTA value proposition”: using simple, story-telling techniques, an easily understood representation of the value HTA should be developed. This value proposition should describe what HTA is (including the notion that it is more than just cost-effectiveness) and what it can be used for.
2. Explore how the INAHTA impact framework and checklists can be expanded to include system level metrics to evaluate the extent to which HTA activities focus on the most pressing issues in health systems and improving overall system efficiency. Explorations could include prioritization processes linked to epidemiology, burden of disease, areas of workforce challenges, or other health system pressures. Consideration could be given to how an expanded framework can be applied in both higher-income and LMIC settings.

GPF participants agreed that HTAi would be an appropriate organization to lead these activities, in partnership with similar organizations, such as INAHTA, particularly for the second priority action. Engaging and consulting multiple stakeholder groups during the processes will be essential. Work is already underway to further these recommendations by HTAi.
